# Yogliptin monotherapy in type 2 diabetes: A 12‐week randomized, double‐blind, placebo‐controlled phase II study

**DOI:** 10.1111/1753-0407.13337

**Published:** 2022-12-14

**Authors:** Xin Wang, Ying Wang, Xiaolan Yong, Bojun Wu, Zilin Sun, Ning Lou, Qing Wen, Yufang Zhang, Shiyun Li, Jiarui Li, Yan He, Jinluo Cheng, Xiangdong Zhong, Jing Shen, Wenying Yang

**Affiliations:** ^1^ China‐Japan Friendship Hospital Beijing China; ^2^ Chengdu Easton Biopharmaceuticals Co., Ltd Chengdu China; ^3^ Chengdu Xinhua Hospital Chengdu China; ^4^ Zhongda Hospital Affiliated to Southeast University Nanjing China; ^5^ Jinan Central Hospital Jinan China; ^6^ Chongqing Red Cross Hospital Chongqing China; ^7^ Affiliated Hospital of Chengdu University Chengdu China; ^8^ Cangzhou Central Hospital Cangzhou China; ^9^ Affiliated Hospital of Guizhou Medical University Guizhou China; ^10^ Changzhou Second People's Hospital Changzhou People's Republic of China; ^11^ Chengdu Fifth People's Hospital Chengdu People's Republic of China

**Keywords:** monotherapy, type 2 diabetes mellitus, xanthine dipeptidyl peptidase‐4 inhibitor, yogliptin, 优格列汀, 单药疗法, 2型糖尿病, DPP‐4抑制剂

## Abstract

**Background:**

The new xanthine dipeptidyl peptidase‐4 inhibitor yogliptin has exhibited excellent hypoglycemic activity in experimental disease models. The present work aimed to assess the efficacy of yogliptin as a monotherapy in individuals with type 2 diabetes mellitus (T2DM).

**Methods:**

A 12‐week, double‐blind, placebo‐controlled phase II study was performed. T2DM patients (new diagnosis or inadequately controlled) were randomly divided into groups (1:1:1:1) and administered either a placebo or weekly doses of 200, 300, or 400 mg yogliptin, respectively. The primary efficacy end point in this analysis was hemoglobin A1c (HbA1c) change at 12 weeks relative to baseline. Relevant secondary outcomes were also examined, including fasting plasma glucose (FPG), 2 h‐postprandial plasma glucose (PPG), body weight, and the rate of individuals who achieved the treatment goal of HbA1c ≤ 7% at 12 weeks from baseline.

**Results:**

A total of 81 cases who received either the placebo (20 cases) or 200 (20 cases), 300 (20 cases), or 400 (21 cases) mg yogliptin were examined in the full analysis set. At 12 weeks, changes in HbA1c levels from baseline were 0.17 (−0.22, 0.57) in the placebo group, and −0.75 (−1.15, −0.35), −0.52 (−0.93, −0.11) and −1.02 (−1.41, −0.64) (mean % [95% confidence interval], *p* < .001 vs. placebo) in the 200, 300, and 400 mg yogliptin groups, respectively. From week four, significant improvements in secondary efficacy outcomes among patients administered the yogliptin monotherapy were observed. FPG showed markedly more pronounced reduction after treatment with yogliptin at 200, 300, and 400 mg in comparison with placebo patients at 4, 8, and 12 weeks. At 12 weeks, goal attainment (HbA1c ≤ 7%) was reached in 0%, 20.00%, 15.80%, and 33.33% of the placebo and three Yogliptin dosage groups, respectively. Adverse events were comparable in all groups.

**Conclusions:**

This study demonstrated that yogliptin controlled glycemia in Chinese T2DM cases, with a great safety profile. The current findings supported that any of the three doses of yogliptin, administered once a week, could be used for phase III clinical studies.

## Graphical abstract

**Highlights**

- Yogliptin at 200, 300, and 400 mg significantly improves glycemic control in diabetics with suboptimal control.
- Yogliptin at all doses was safe in type 2 diabetic patients.

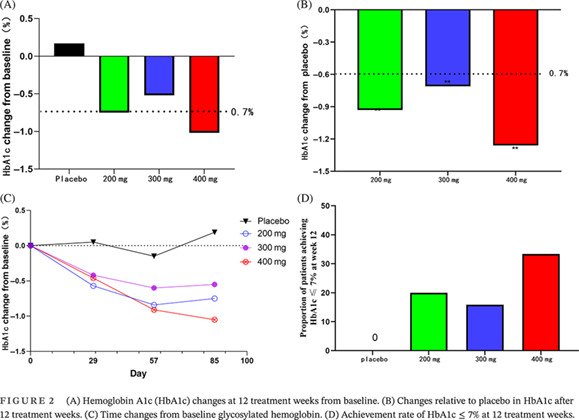

## 摘要

背景与目的:新型DPP‐4抑制剂优格列汀在实验性疾病模型中显示出良好的降糖活性。本研究旨在评估优格列汀单药治疗2型糖尿病(T2DM)的疗效。

方法:本研究为一项为期12周、双盲、安慰剂对照的II期研究。2型糖尿病(T2DM)患者(新诊断或血糖控制不佳)随机(1∶1∶1∶1)分组, 分别给予安慰剂或每周200、300或400 mg 优格列汀。此项分析的主要疗效终点是12周时HbA_1c_相对于基线的变化。同时检测了相关的次要结局指标, 包括FPG、2h‐PPG、体重以及12周时HbA_1c_达标率(HbA_1c_≤7%)。

结果:共81例患者接受安慰剂(20例)、200 mg(20例)、300 mg(20例)和400 mg(21例)优格列汀治疗, 并进入全分析集(FAS)。12周时, 安慰剂组的HbA_1c_水平相对于基线的变化为0.17(‐0.22,0.57), 200 mg、300 mg和400 mg 优格列汀组分别为‐0.75(‐1.15, ‐0.35)、‐0.52(‐0.93, ‐0.11)和‐1.02(‐1.41, ‐0.64)(中位% (95% CI), P<0.001 vs安慰剂)。从第4周开始, 接受优格列汀单药治疗的患者次要疗效结局有显著改善。在第4、8和12周时, 200 mg、300 mg和400 mg 优格列汀组的FPG下降幅度均显著高于安慰剂组。12周时, 安慰剂组和优格列汀3个剂量组的达标率(HbA_1c_≤7%)分别为0%、20.00%、15.80%和33.33%。各组的不良事件相当。

结论:优格列汀可有效控制中国2型糖尿病患者的血糖, 且安全性良好。目前的研究结果表明, 优格列汀每周给药1次的3种剂量中的任何一种均可用于III期临床研究。

## 1 INTRODUCTION

Worldwide, an estimated 387 million people live with diabetes mellitus.^1^ In China, diabetes represents a major chronic disease affecting 113.9 million adults, making China the nation with the largest number of diabetics.^2^ The American Diabetes Association (ADA) and the International Diabetes Federation (IDF) propose an optimal glycemic target for hemoglobin A1c (HbA1c) approximating ≤7.0% in the majority of individuals with diabetes.^1^, ^3^, ^4^ Meanwhile, IDF, the American Association of Clinical Endocrinologists,^4^, ^5^ and ADA have proposed a stricter HbA1c goal (6.5%) in case this is achievable with no pronounced hypoglycemia or other adverse events (AEs).^3^ However, despite these recommendations, optimal glycemic control has become an elusive target in multiple individuals.

The clinical application of dipeptidyl peptidase‐4 (DPP‐4) suppressors has gained traction recently. Experts currently recommend DPP‐4 inhibitors for first‐line therapy in individuals not tolerating metformin, especially the elderly and underweight individuals, because these products do not affect body weight and rarely induce hypoglycemia.^6^ Clinical studies have assessed the efficacy of multiple DPP‐4 inhibitors in type 2 diabetes mellitus (T2DM). Indeed, DPP‐4 inhibitors are increasingly used to manage T2DM.^7^ Yogliptin represents a newly discovered xanthine DPP‐4 inhibitor, which reversibly and covalently binds to DPP‐4, with a low dissociation rate. Like other known agents that inhibit DPP‐4, yogliptin has a >100‐fold higher affinity for DPP‐4 compared with DPP‐8 or DPP‐9. Preclinical experiments and a phase I trial have revealed yogliptin effectively and dose‐dependently inhibits DPP‐4 activity, with an average ≥80% reduction in plasma DPP‐4 activity over 168 h following a single dose of yogliptin at ≥100 mg.^8^ No dose‐limiting toxicity occurred in healthy subjects at 2.5–600 mg. At the same time, in comparison to existing DPP‐4 inhibitors, yogliptin characteristically alleviates allergic reactions, improves bioavailability, enhances efficacy, and has unique intestinal excretion.^8^ Database assessment of prescription records and individual preferences indicate efficacious and safe antihyperglycemic agents administered once a week may help increase treatment adherence in diabetics. Moreover, compliance is an important factor affecting glycemic control; thus, as a weekly preparation, yogliptin is simple and convenient and could help improve patient compliance.

A multicenter, randomized, double‐blind, placebo‐controlled phase II study was carried out to assess yogliptin's efficacy as a monotherapy in diabetes.

## 2 METHODS

### 2.1 Patients

Inclusion criteria were: T2DM diagnosed within 24 months based on the 1999 World Health Organization diagnostic criteria for diabetes mellitus: age between 18 and 70 years, no regular antihyperglycemic drug administered for ≥8 weeks, body mass index of 19–30 kg/m^2^, and HbA1c of 7.0%–10.0%. Exclusion criteria were fasting plasma glucose (FPG) >13.9 mM; systolic (SBP) and diastolic (DBP) blood pressure >160 and >100 mmHg, respectively; impaired liver function, with aspartate transaminase and/or alanine transaminase amounts increasing at least twofold beyond the upper limit of normal range; reduced renal function, with glomerular filtration rate ≤60 ml/min/1.73 m^2^; second‐ or third‐degree atrioventricular block, with long QT syndrome (QTc >500 ms on a 12‐lead electrocardiogram [ECG] with no pacemaker); triglyceride levels >5.6 mM; enrollment in other studies within 3 months; and pregnancy or lactation in women. Individuals with acute diabetic complications, insulin requirement, glucocorticoid administration within 14 days before enrolment, and/or other conditions unsuitable for this trial according to investigators were also excluded.

This trial followed good clinical practice guidelines and the Declaration of Helsinki and had approval from the ethics committees of various centers involved. Signed informed consent was provided by each patient before enrolment.

### 2.2 Study design

This was a multicenter, randomized, double‐blind, placebo‐controlled, parallel‐group phase II study evaluating weekly yogliptin (tablet form) administration for its efficacy as a monotherapy in diabetic patients. In total, 13 centers in China were involved, and the study ran from 30 May 2019 to 31 May 2021. The trial underwent registration at www.chictr.org.cn (ChiCTR2100050592). Prospective participants were submitted to 2‐week screening, 4‐week lead‐in (controlling diet and exercise), and 1‐week baseline evaluation periods pre‐randomization. Eligible individuals underwent randomization (1:1:1:1) and received the placebo or weekly doses of yogliptin at either 200, 300, or 400 mg, respectively, for 12 weeks with a 2‐week follow‐up at treatment end. Random numbers were generated with an Interactive Web Response System. Participants, the medical staff, and investigators were blinded to treatment allocation.

## 3 STUDY DRUG

Yogliptin tablet was proposed at a 200‐mg single weekly dose (qw) for T2DM in clinic. Thus, the 200, 300, and 400 mg yogliptin weekly doses were comparatively assessed against the placebo. In this trial, 100 mg yogliptin tablets were applied versus matching placebo tablets. The yogliptin 200 mg group patients were administered two 100 mg yogliptin and two placebo tablets. The yogliptin 300 mg group was administered three 100 mg yogliptin and one placebo tablet. The yogliptin 400 mg group was administered four yogliptin 100 mg tablets. The placebo group was administered four placebo tablets. Oral administration was applied in all cases.

### 3.1 Efficacy and safety outcomes

The primary efficacy endpoint was HbA1c change at 12 weeks from baseline. Secondary efficacy endpoints were plasma‐activated glycated albumin, FPG, 2‐h postprandial plasma glucose (PPG), and body weight changes at 12 weeks relative to baseline values, as well as the rate of patients achieving the goal of HbA1c ≤ 7% at 12 weeks.

Safety parameters included the rates and severity of AEs, vital sign anomalies, 12‐lead ECG changes, and laboratory test data for hematology, urinalysis, and blood chemistry. AE severity was classified as follows: (1) no or mild symptoms, requiring no intervention; (2) moderate, limited effects on age‐appropriate daily live activities, requiring minimal, local noninvasive interventions; and (3) severe, pronounced effects on self‐care or daily living activities but no immediate life‐threatening effects, requiring hospital admission or prolonged hospitalization.

Plasma glucose ≤3.9 mM constituted a threshold for hypoglycemic events, even without symptoms. Sweating, heart palpitations, shaking, dizziness and hunger were also considered hypoglycemic events. Each case was required to report any suspected hypoglycemic event for proper treatment.

### 3.2 Statistical analysis

One meta‐analysis^9^, ^10^ showed a combined effect size (MD) reduction of glycated hemoglobin of −0.38% for alogliptin; another meta‐analysis showed that the MD for the DPP‐4‐week formulation (aulogliptin and trelagliptin) was −0.63%, 95% confidence interval (CI) [−0.80, −0.46]. Based on literature analysis, the difference in HbA1c change from baseline at 12 weeks between the eudagliptin tablet group and the placebo group was estimated to be −0.4%. When the significance level is set at 0.2 on both sides and the power is set at 70%, considering the dropout rate of 20%, it is estimated that a total of 80 subjects would need to be included in the present study, with 20 subjects in each group. SAS® 9.4 (SAS Institute, USA) was utilized for data analysis, with *p* < 0.05 indicating statistical significance. All randomized individuals administered one or more doses of the study drug, with available post‐therapy data, were included in the full analysis set (FAS). Baseline features, as well as efficacy and safety end points, were analyzed based on the FAS. Continuous data were represented as mean ± SD. Analysis of variance or a nonparametric test was utilized for comparisons according to data distribution. The Dunnett test was performed to assess treatment groups versus patients administered placebo. Paired *t* test or a nonparametric test was performed to compare changes from baseline in individual groups. Categorical data were presented as count and ratio, and group pairs were compared by the chi‐square test or Fisher's exact test. Nominal data were assessed using the rank sum test. The rates of AEs were assessed using the Fisher's exact test.

## 4 RESULTS

**FIGURE 1:**
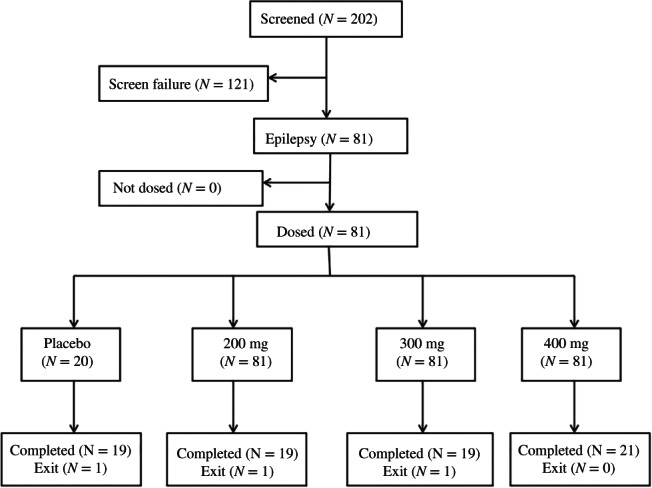
Study flow chart.

In total, 202 individuals were enrolled, and 81 underwent randomization (Figure 1). The top reason for exclusion was unmet HbA1c entry levels, followed by unmet laboratory result requirements. A total of three (3.7%) patients discontinued the study, and 78 individuals completed the whole study. Table 1 shows that demographics, anthropometric features, disease properties, and efficacy outcomes were similar among groups at baseline. In the whole patient population, the mean T2DM course and HbA1c levels were 1.21 years and 8.36%(Table 1), respectively. The frequencies and types of baseline medical conditions were similar in various groups. as well as concomitant medications.

**TABLE 1:** Baseline demographics, anthropometric features, disease characteristics, and efficacy end point data in the whole patient population

Parameter	Placebo (*N* = 20)	200 mg (*N* = 20)	300 mg (*N* = 20)	400 mg (*N* = 21)		*p* value
Age (years)	55.6 ± 9.54	54.4 ± 11.32	51.1 ± 10.65	51.7 ± 10.86	0.83	.4798
Body weight (kg)	69.86 ± 11.057	67.94 ± 9.378	70.07 ± 13.381	69.15 ± 12.004	0.14	.9366
Body mass index (kg/m^2^)	25.76 ± 2.624	25.16 ± 2.076	25.73 ± 2.825	25.92 ± 2.256	0.37	.7728
Male, *n* (%)	14 (70.0%)	13 (65.0%)	13 (65.0%)	10 (47.6%)		.4980
T2DM course (years)	1.34 ± 0.85	1.22 ± 0.71	0.93 ± 0.53	1.38 ± 0.88	1.43	.2401
Han race, *n* (%)	20 (100%)	20 (100%)	20 (100%)	21 (100%)		
Glycated hemoglobin A1c (%)	8.34 ± 0.741	8.25 ± 0.607	8.48 ± 0.730	8.36 ± 0.439	0.43	.7301
Fasting plasma glucose (mmol/L)	9.976 ± 2.1210	9.879 ± 1.3628	10.823 ± 2.3823	10.271 ± 1.92	0.92	.4350
Systolic blood pressure (mmHg)	102.7 ± 29.42	99.9 ± 28.54	108.0 ± 27.38	98.8 ± 22.96	0.46	.7106
Diastolic blood pressure (mmHg)	103.9 ± 23.84	100.1 ± 24.83	108.4 ± 33.60	103.0 ± 28.26	0.30	.8222

Abbreviation: T2DM type 2 diabetes mellitus.

*N*: Number of subjects in each group.

### 4.1 Primary efficacy outcomes

**FIGURE 2:**
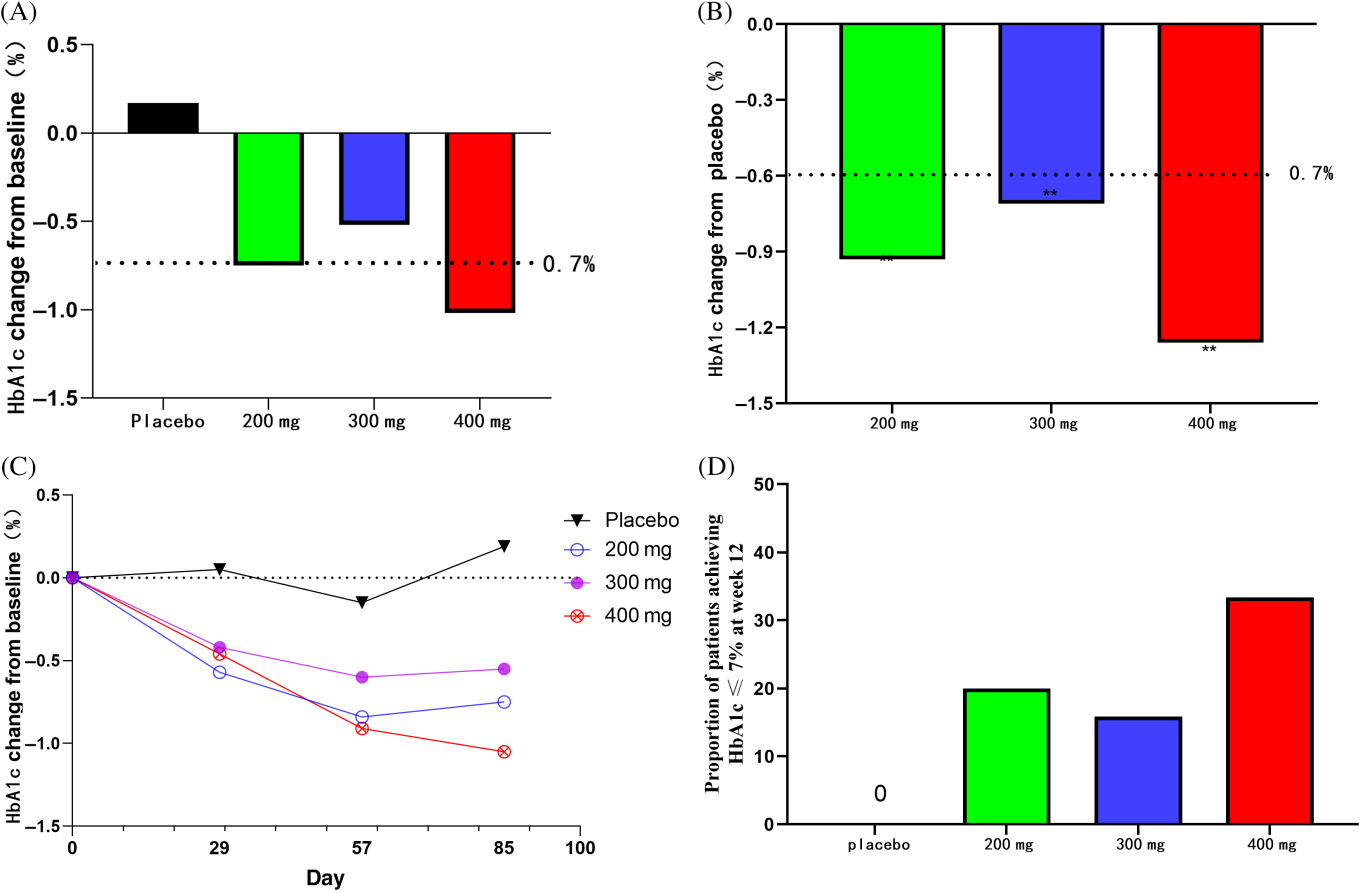
(A) Hemoglobin A1c (HbA1c) changes at 12 treatment weeks from baseline. (B) Changes relative to placebo in HbA1c after 12 treatment weeks. (C) Time changes from baseline glycosylated hemoglobin. (D) Achievement rate of HbA1c ≤ 7% at 12 treatment weeks.

HbA1c changes at 12 weeks from baseline (%, means [95% CI]) were 0.20 (−0.23， 0.62) in the placebo group and −0.78 (−1.19， −0.36), −0.52 (−0.94， −0.10) and −1.06 (−1.46， −0.65) in yogliptin 200, 300, and 400 mg groups, respectively (*p* < .05 vs. placebo; Figure 2 and Table 2). HbA1c changes at 12 weeks showed no marked differences between the yogliptin groups.

**TABLE 2:** Effects of yogliptin on hemoglobin A1c (HbA1c), fasting plasma glucose (FPG), 2 h‐postprandial plasma glucose (PPG), body weight, and HbA1c ≤ 7% at 12 weeks

Eifficacy Parameter		Placebo (*N* = 20)	Yogliptin 200 mg (*N* = 20)	Yogliptin 300 mg (*N* = 20)	Yogliptin 400 mg (*N* = 21)
HbA1c	LS mean change from baseline	0.20 (−0.23,0.62)	−0.78 (−1.19, −0.36)	−0.52 (−0.94, −0.10)	−1.06 (−1.46, −0.65)
	Difference in means (Yogliptin–placebo [95% CI])	‐	−0.97 (−1.57, −0.38)	−0.71 (−1.31, −0.12)	−1.26 (−1.84, −0.67)
FPG	LS mean change from baseline	0.38 (−0.67, 1.44)	−1.06 (−2.09, −0.03)	−1.59 (−2.63, −0.55)	−1.30 (−2.30, −0.30)
	Difference in means (Yogliptin–placebo [95%CI])	‐	−1.44 (−2.92, 0.04)	−1.98 (−3.45, −0.50)	−1.68 (−3.14, −0.23)
2‐h‐PPG	Mean ± SD	17.30 ± 4.52	15.73 ± 2.75	16.44 ± 2.66	14.11 ± 3.12
Weight	*t* value	−1.10	1.73	1.41	0.33
	*p* value	.29	.10	.18	.74
HbA1c ≤ 7%	%	0	21.10%	15.80%	35.00%

Abbreviations: CI, confidence interval; LS, Least squares.

### 4.2 Secondary efficacy outcomes

FPG, 2 h‐PPG, body weight, and the rate of individuals who achieved the treatment goal of HbA1c ≤ 7% at 12 weeks from baseline were examined.

**FIGURE 3:**
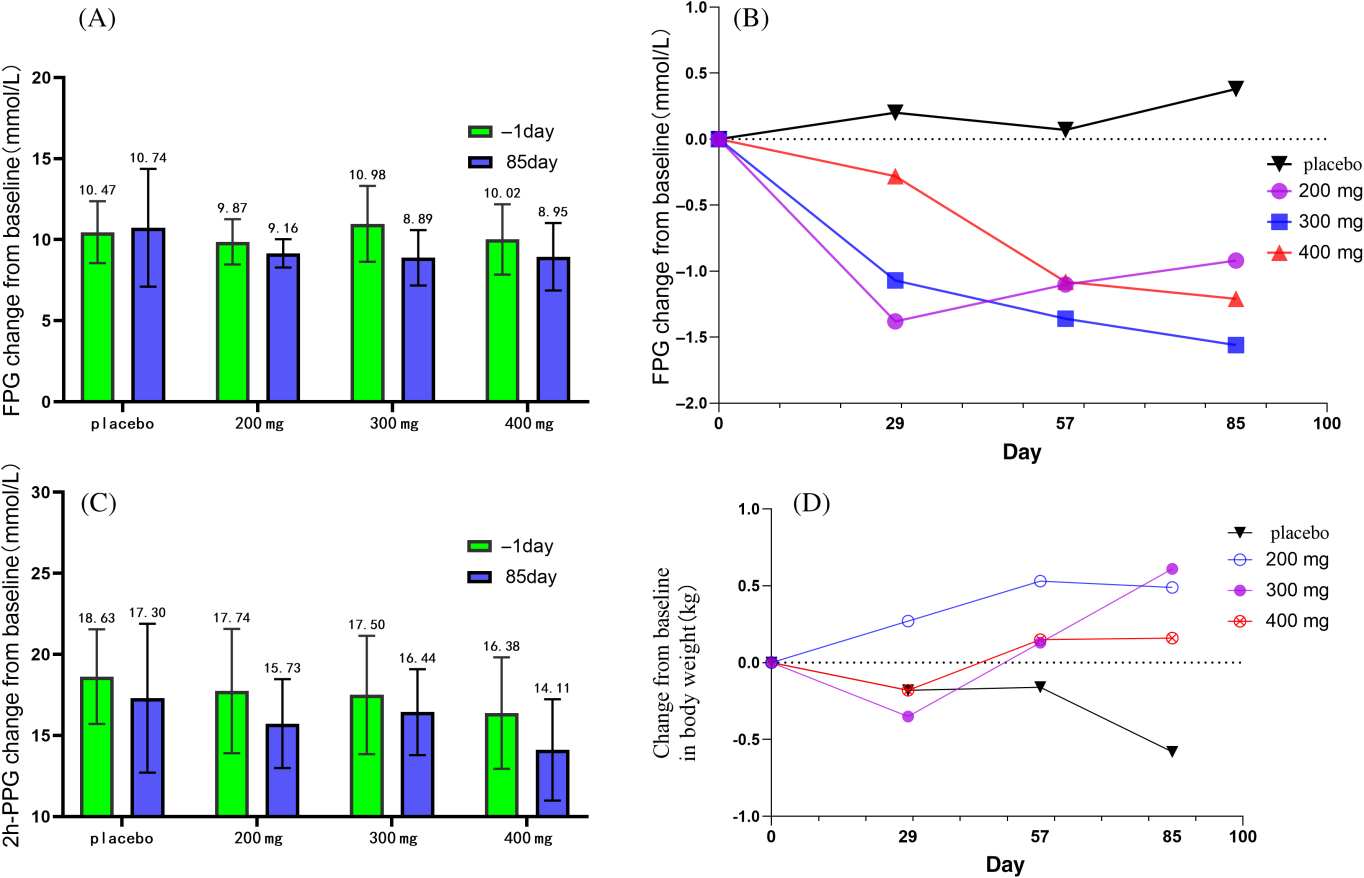
(A) Fasting plasma glucose (FPG) changes at 12 weeks of treatment from baseline. (B) Time changes in FPG. (C) 2 h‐postprandial plasma glucose (PPG) changes at 12 treatment weeks from baseline. (D) Time changes from baseline of weight.

At 12 weeks, the rates of individuals who achieved the goal of HbA1c ≤ 7% were 0%, 21.10%, 15.80%, and 35.00% in the placebo and 200, 300, and 400 mg yogliptin groups, respectively (Figure 3 and Table 2).

The reduction in FPG from baseline was markedly higher post‐treatment with yogliptin at 200, 300, and 400 mg compared with the placebo group at 12 weeks (Figure 3A and Table 2). All yogliptin doses reduced 2‐h PPG at week 12; however, 2‐h PPG values at baseline were similar between all four groups. At 12 weeks, body weight changes from baseline were comparable among the yogliptin and placebo groups (Figure 3D).

### 4.3 Safety

The rates of individuals with AEs, serious adverse events (SAEs) and drug‐related AEs were similar in all groups, as were the rates of AEs requiring treatment discontinuation (Table 3). No hypoglycemia occurred in the placebo or yogliptin 200, 300, and 400 mg groups. In total four cases (*N* = 5 events) had SAEs. Among them, four events in four cases had no associations with the drug, whereas one event in one case was drug related. In the yogliptin 200 mg group, one patient was considered to have developed liver dysfunction due to elevated liver enzyme indicators. The patient was definitely diagnosed with primary sclerosing cholangitis during subsequent analysis, and liver dysfunction was considered drug independent.

**TABLE 3:** Summary of adverse events

Adverse events during the trial	Placebo (*N* = 20)	200 mg (*N* = 20)	300 mg (*N* = 20)	400 mg (*N* = 21)	Total (*N* = 81)
Adverse events (AEs)	13 (65.0%)	15 (75.0%)	18 (90.0%)	16 (76.2%)	62 (76.5%)
Drug‐related AEs	8 (40.0%)	10 (50.0%)	9 (45.0%)	6 (28.6%)	33 (40.7%)
Severe AEs	0	0	0	0	0
Serious AEs	1 (5.0%)	1 (5.0%)	1 (5.0%)	1 (4.8%)	4 (4.9%)
Drug‐related serious AEs	0	1 (5.0%)	0	0	1 (1.2%)
AEs leading to withdrawal	1 (5.0%)	1 (5.0%)	1 (5.0%)	0	3 (3.7%)
Severity of adverse event					
Level 1	7 (35.0%)	10 (50.0%)	14 (70.0%)	14 (66.7%)	45 (55.6%)
Level 2	4 (20.0%)	3 (15.0%)	1 (5.0%)	1 (4.8%)	9 (11.1%)
Level 3	2 (10.0%)	2 (10.0%)	3 (15.0%)	1 (4.8%)	8 (9.9%)
Level 4	0	0	0	0	0
Level 5	0	0	0	0	0
Missing	0	0	0	0	0

*Note*: An adverse reaction was defined as an adverse event that was certainly, probably, or possibly associated with the study drug. Significant adverse events are hypoglycemic events.

The AEs that occurred in >5% of cases included hyperuricemia, lipid metabolism disorder, urinary tract infection, and hyperlipidemia. The majority of drug‐related AEs had comparable rates in the four groups.

The percentages of cases meeting predefined limits of change (PDLC) criteria did not significantly change from baseline for laboratory parameters. Mean PDLC changes were mostly low and similar in the yogliptin and placebo groups. Pulse rate, SBP, DBP, and weight changes were similar among the four groups as well.

## 5 DISCUSSION

The efficacy of yogliptin in T2DM patients was assessed by comparing orally administered yogliptin at 200, 300, and 400 mg, respectively, with the placebo group. According to the results, yogliptin markedly decreased plasma glucose amounts at all doses (200, 300, and 400 mg) in comparison with placebo administration from 4 to 12 weeks. Besides this, yogliptin improved HbA1c, FPG, and PPG in comparison with placebo. However, the effects of yogliptin on FPG showed no significant dose dependence between the three dose groups. There were 81 subjects in this study. Among them, 20 subjects received 300 mg and 19 subjects completed the trial. The sample size was small, which led to the possibility of lower HbA1c in the 300 mg group only. At the same time, 2 h‐PPG glucose changes in the 300 mg were less than those in the 200 and 400 mg groups, resulting in a lower glycated hemoglobin control effect. This represents an overall limitation of the study regarding sample size as the sample number may have skewed results between individual groups but did not affect the overall result trend, but does indicate the need for further studies. Furthermore, there were no major safety concerns associated with the use of yogliptin for 12 weeks, and the incidence rates of AEs did not increase in a dose‐dependent manner across the doses tested (200–400 mg).

National guidelines have included DPP4 inhibitors in treatment algorithms for managing T2DM based on the finding that DPP4 clearly plays a vital role in scavenging a potent incretin hormone, glucagon‐like peptide 1.^11^, ^12^, ^13^, ^14^ DPP‐4 inhibitors can not only reduce blood glucose but also decrease fasting blood glucose. Several clinical studies^15^, ^16^ have revealed DPP‐4 suppressors can decrease fasting blood glucose by 0.5–1.0 mM and 2‐h blood glucose by 2.0–3.0 mM after removing the placebo effect. After removing the placebo effect, DPP‐4 inhibitors reduced HbA1C by about 0.5% ~ 0.9% (mean decrease of 0.7%), depending on baseline HbA1C levels. These findings suggest DPP‐4 inhibitors decrease blood glucose and absolute HbA1C.

After 12 weeks of treatment with yogliptin, taking into account the placebo effect, FPG changes in the 200, 300, and 400 mg yogliptin groups were −1.12, −1.76, and −1.41 mmol/L, respectively; 2‐h blood glucose reduction rates were 0, −0.71, and −0.94 mmol/L, respectively, and the HbA1C reduction rates were −0.93%, −0.69%, and −1.20%, respectively. Generally, fasting postprandial and postprandial after 2 weeks of yogliptin administration at 200, 300, and 400 mg were similar to values obtained for other DPP‐4 target drugs. DDP‐4 drugs had no significant effect on body weight. In this trial, after 12 weeks of administration, the overall body weight of the treatment group did not change significantly, and the change range was less than 1 kg. The results were consistent with other DPP‐4 drugs.^17^

As shown here, yogliptin was well tolerated in the totality of groups for 12 weeks and did not induce overt AEs. Drug‐related AEs included mild or moderate hyperuricemia, lipid metabolism disorder, urinary tract infection, and hyperlipidemia. The rates of hypoglycemia were low and similar among all groups. It has been reported that α‐cells exhibit higher responsiveness to low ambient glucose concentrations with the help of DPP‐4 inhibitors.^18^ The Food and Drug Administration cautioned that DPP‐4 suppressors may increase the risk of serious arthralgia,^19^ attracting great attention, revealing 33 serious arthralgia cases after using DPP‐4 inhibitors over a 7‐year period, but no case developed arthralgia in the yogliptin treatment groups in this research. Optimal adherence to oral antidiabetics is achieved in approximately one third of all diabetics. This may result from prolonged treatment, regimen complexity, and treatment frequency, among other reasons. The lower the dosing frequency, the better the adherence to antidiabetic treatment. Therefore, a drug administered weekly may show high adherence, providing a great option for diabetics.

At present, no weekly DPP‐4 inhibitor is marketed in China. Here, 99% or more of the patients complied with the whole treatment, which indicates a strength of the study and potential longer‐term compliance with treating patients using this technique. However, the study was performed for only 12 weeks, which represents a study limitation; therefore, yogliptin safety should be further assessed in long‐term studies.

## 6 CONCLUSIONS

Yogliptin as a monotherapy controlled glycemia in Chinese T2DM cases, with good safety profile. These findings supported all three doses of yogliptin administered once a week could be chosen for phase III clinical studies of diabetes.

## DISCLOSURE

The authors have no conflict of interest.
